# Stable isotopic labelling-assisted untargeted metabolic profiling reveals novel conjugates of the mycotoxin deoxynivalenol in wheat

**DOI:** 10.1007/s00216-012-6483-8

**Published:** 2012-10-20

**Authors:** Bernhard Kluger, Christoph Bueschl, Marc Lemmens, Franz Berthiller, Georg Häubl, Günther Jaunecker, Gerhard Adam, Rudolf Krska, Rainer Schuhmacher

**Affiliations:** 1 Center for Analytical Chemistry, Department for Agrobiotechnology (IFA-Tulln), University of Natural Resources and Life Sciences (BOKU), Vienna, Konrad-Lorenz-Str. 20, 3430 Tulln, Austria; 2 Institute for Biotechnology in Plant Production, Department for Agrobiotechnology (IFA-Tulln), University of Natural Resources and Life Sciences (BOKU), Vienna, Konrad-Lorenz-Str. 20, 3430 Tulln, Austria; 3 Romer Labs Diagnostic GmbH, Technopark 1, 3430 Tulln, Austria; 4 Department of Applied Genetics and Cell Biology, University of Natural Resources and Life Sciences (BOKU), Vienna, Konrad-Lorenz-Str. 24, 3430 Tulln, Austria

**Keywords:** Metabolisation, Xenobiotics, Stable isotopic labelling, Deoxynivalenol, Liquid chromatography–high resolution mass spectrometry, Mycotoxin conjugate

## Abstract

An untargeted screening strategy for the detection of biotransformation products of xenobiotics using stable isotopic labelling (SIL) and liquid chromatography–high resolution mass spectrometry (LC-HRMS) is reported. The organism of interest is treated with a mixture of labelled and non-labelled precursor and samples are analysed by LC-HRMS. Raw data are processed with the recently developed MetExtract software for the automated extraction of corresponding peak pairs. The SIL-assisted approach is exemplified by the metabolisation of the *Fusarium* mycotoxin deoxynivalenol (DON) *in planta*. Flowering ears were inoculated with 100 μg of a 1 + 1 (*v*/*v*) mixture of non-labelled and fully labelled DON. Subsequent sample preparation, LC-HRMS measurements and data processing revealed a total of 57 corresponding peak pairs, which originated from ten metabolites. Besides the known DON and DON-3-glucoside, which were confirmed by measurement of authentic standards, eight further DON-biotransformation products were found by the untargeted screening approach. Based on a mass deviation of less than ±5 ppm and MS/MS measurements, one of these products was annotated as DON-glutathione (GSH) conjugate, which is described here for the first time for wheat. Our data further suggest that two DON-GSH-related metabolites, the processing products DON-*S*-cysteine and DON-*S*-cysteinyl-glycine and five unknown DON conjugates were formed *in planta*. Future MS/MS measurements shall reveal the molecular structures of the detected conjugates in more detail.

## Introduction

Xenobiotics are frequently metabolised and subsequently conjugated to more polar derivatives as a part of detoxification strategies of organisms, including animals and plants. For the determination of metabolic pathways, mass spectrometry turned out to be one of the most powerful techniques for the detection of all types of low-molecular weight metabolites even on systems level [1–3]. In metabolomics, targeted approaches aim at the quantification of known or predicted specific metabolites, while untargeted approaches try to probe the global metabolic space of a biological sample [4]. As a major advantage over targeted methods, untargeted metabolomics approaches have the potential to discover novel and unexpected metabolites such as unknown biotransformation products originating from specific xenobiotics.

Stable isotopic labelling (SIL)-assisted metabolomics techniques [5–9] offer a wide range of new applications in the field of untargeted approaches. In vivo SIL for example can be used for the global detection of biologically derived metabolite signals and offers a high degree of certainty that detected MS signals represent actual metabolites and are not resulting from e.g. solvents, matrix background or noise. Moreover, SIL-assisted approaches provide additional information on the number of carbon atoms of the detected metabolite ions [5]. Both stable isotopic labelled endogenous [10] as well as xenobiotic [3, 11] precursors have been used to study in vivo metabolisation in biological samples. In this context, a major challenge is the detection of the labelled biotransformation products within highly complex liquid chromatography–high resolution mass spectrometry (LC-HRMS) chromatograms. The recently developed software algorithm MetExtract [12] is capable of the automated global detection of metabolite-derived LC-HRMS signals originating from non-labelled and stable isotopically labelled compounds.

Here, we present the first results of a SIL-assisted metabolomics approach for the untargeted screening of xenobiotics and its derived biotransformation products exemplified by the metabolisation of the *Fusarium graminearum* mycotoxin deoxynivalenol (DON). DON [13] belongs to the group of trichothecene mycotoxins and is a frequent contaminant of food and feed. It is produced by *F*. *graminearum* during infection of cereal grains such as wheat and barley [14]. The protein synthesis inhibitor DON is a virulence factor of *Fusarium*, and in turn, detoxification of DON seems to be an important component of *Fusarium* resistance in wheat [15, 16]. Plants can reduce the toxicity of DON by conjugation of the toxin to glucose [17]. In this study, we applied a SIL-assisted approach for the untargeted screening of biotransformation products of DON in wheat. We found eight novel wheat-derived DON conjugates and for the first time obtained clear evidence that glutathione-mediated metabolism of DON occurs in wheat.

## Experimental section

### Chemicals and reagents

Methanol (MeOH, LiChrosolv, LC gradient grade) was purchased from Merck (Darmstadt, Germany); acetonitrile (ACN, HiPerSolv Chromanorm, HPLC gradient grade) was purchased from VWR (Vienna, Austria); formic acid (FA, MS grade) was obtained from Sigma-Aldrich (Vienna, Austria). Water was purified successively by reverse osmosis and an ELGA Purelab Ultra-AN-MK2 system (Veolia Water, Vienna, Austria). Non-labelled DON and uniformly labelled ^13^C_15_ DON were obtained as a contribution for that particular study from Romer Labs (Tulln, Austria) in crystalline form, not commercially available. DON-3-glucoside (D3G) standard was produced according to [18].

### Standard preparation

Standard solutions of DON and D3G with concentrations of 1 mg L^−1^ were prepared in methanol/water (1 + 1, *v*/*v*) and used as analytical standards. Lyophilised non-labelled DON and ^13^C_15_-labelled DON for inoculation of wheat were dissolved separately in pure water to a concentration of 1 g L^−1^. Both stock solutions were mixed 1 + 1 (*v*/*v*) to obtain an inoculation solution containing 500 mg L^−1^ non-labelled DON + 500 mg L^−1^ ^13^C_15_ DON.

### Treatment of wheat samples

Wheat plants (cultivar “Remus”, which is sensitive for both Fusarium head blight and DON) were grown under standardised conditions [15]. Flowering ears were either treated with the DON inoculation solution or with water (mock) according to the following procedure: at time point zero, 10 μL DON were injected in each of two adjacent spikelets in the lower part of a flowering ear. Twenty-four hours later, the next two adjacent spikelets, located immediately above those previously treated, were injected with the same amount of toxin. This procedure was repeated 48, 72 and 96 h after the first treatment resulting in a total applied amount of 100 μg DON per ear. All treatments were carried out in triplicate. One hundred and eight hours after the first inoculation, the ear bases, containing all inoculated spikelets of DON-treated and mock ears, were sampled separately and immediately shock-frozen in liquid nitrogen. That way, we aimed to target all DON metabolites present *in planta* between 12 to 108 h post-treatment. All samples were stored at −80 °C until further sample preparation.

### Sample preparation

Frozen wheat ears were milled separately to a fine powder for 2 min at 30 Hz using a ball mill (MM301 Retsch, Haan, Germany) with pre-cooled (liquid nitrogen) 10-mL stainless steel vessels (Retsch) and a 9-mm stainless steel ball (Retsch). Of homogenised wheat material, 105 ± 5 mg was weighed to 1.5-mL Eppendorf tubes and extracted with 1 mL of pre-cooled methanol/water 3 + 1 *v*/*v* including 0.1 % formic acid, by vortexing for 10 s, and subsequent treatment in an ultrasonic bath at room temperature for 15 min according to de Vos et al. [19]. Samples were centrifuged for 4 min at 8,500×*g* (9,500 rpm) at room temperature. An aliquot of 600 μL supernatant was transferred to another 1.5-ml Eppendorf tube adding 300 μL water + 0.1 % formic acid to achieve a final methanol/water ratio of 1:1 (*v*/*v*). All samples were vortexed for 10 s before transfer into HPLC vials for LC-MS measurements.

### LC-HR-MS conditions

LC-HR-MS measurements were performed on an LTQ Orbitrap XL (Thermo Fisher Scientific) equipped with an electrospray ionisation (ESI) source coupled to an UHPLC system (Accela, Thermo Fisher Scientific, San Jose, CA, USA). The analytical column was a reversed-phase XBridge C_18_, 150 × 2.1 mm i.d., 3.5 μm particle size (Waters, Milford, MA, USA), preceded by a C_18_ 4 × 3 mm i.d. security cartridge (Phenomenex, Torrance, CA, USA). The column temperature was maintained at 25 °C. Eluent A was water and eluent B was MeOH, both containing 0.1 % formic acid. The chromatographic method held the initial mobile phase composition (10 % B) constant for 2 min, followed by a linear gradient to 100 % B within 30 min. This final condition was held for 5 min, followed by 8 min of column re-equilibration at 10 % B. The flow rate of the mobile phase was 250 μL min^−1^ and the injection volume was 10 μL.

The ESI interface was used in positive ion mode with the following settings: sheath gas, 60 arbitrary units; auxiliary gas, 15 arbitrary units; sweep gas, 5 arbitrary units; capillary voltage, 4 kV; capillary temperature, 300 °C. All other source parameters were automatically tuned for maximum signal intensity of a 10-mg L^−1^ reserpine solution (Sigma-Aldrich). For the FT-Orbitrap, the automatic gain control was set to a target value of 3 × 10^5^, and a maximum injection time of 500 ms was chosen. The mass spectrometer was operated in a scan range from *m*/*z* 100 to *m*/*z* 1,000 with a resolving power setting of 60,000 FWHM (at *m*/*z* 400). Data were recorded using Xcalibur 2.1.0 (Thermo Fisher Scientific). For MS/MS measurements, collision-induced dissociation (*Q* = 0.250, activation time 30 ms, resolving power setting 15,000 FWHM) was used.

### Data processing and annotation of DON conjugates

An improved yet unpublished version of MetExtract [12], developed by us, was applied to automatically extract corresponding MS peak pairs in mass spectra of a 1 + 1 mixture of biological samples containing natural and ^13^C fully labelled xenobiotics. Putative DON-metabolisation products had to fulfil the following criteria: (1) the monoisotopic non-labelled and the completely labelled isotopologues of DON-derivative ions form the principal ions of their corresponding isotopic patterns and have to be present in at least two mass spectra, (2) peak area ratio of monoisotopic peaks and corresponding fully labelled analogues has to be 0.8–1.2 and (3) monoisotopic non-labelled and the completely labelled isotopologue ions have to show chromatographic co-elution.

Furthermore, within all LC-HRMS chromatograms, putative DON-derived ion signals were grouped according to retention time with the aim to deconvolute mass spectra and evaluate the number of different metabolites as well as type of ion species.

Different metabolites were characterised and annotated according to following criteria: (1) the accurate mass differs less than ±5 ppm from the theoretical postulated mass and (2) possible heteroatoms within the conjugates (e.g. sulphur) are determined by evaluating the isotopic pattern of the completely labelled isotopologue. Moreover, MS/MS spectra were recorded and evaluated using Thermo Xcalibur 2.1.0.

## Results and discussion

### Detection and characterisation of DON-biotransformation products

**Fig. 1:**
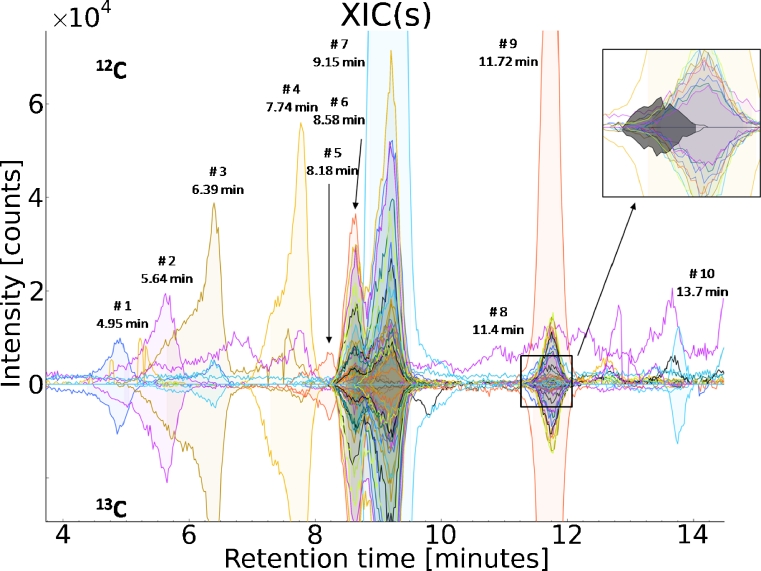
EICs extracted by MetExtract: positive intensities show the EICs of the monoisotopic ^12^C labelled metabolite ions, while negative intensities represent the EICs of the corresponding ^13^C fully labelled metabolite ions. All of the *m/z* values labelled with retention time and number showed similar peak area, peak shape and perfect co-elution and thus can be regarded as signals originating from biotransformation products of DON (*#2* DON-S-Cys, *#3* DON-S-Cys-Gly, *#4* DON-GSH, *#6* DON, *#7* D3G)

MetExtract data processing revealed a total of 57 ion pairs in full-scan chromatograms of DON-treated samples that showed the monoisotopic non-labelled and the corresponding completely labelled isotopologue of the DON moiety. No ion pairs could be detected in mock-treated samples. The obtained features were sorted according to retention time and grouped into ten distinct chromatographic peak groups with the aid of MetExtract. DON (*m*/*z* 297.1327, [M+H]^+^) and nine DON-biotransformation products were detected in wheat samples treated with a 1 + 1 mixture of native and ^13^C_15_-labelled DON. The corresponding peak pairs of all detected DON-biotransformation products showed a mass difference of exactly 15.0503 Da indicating the presence of all 15 carbon atoms of DON. Thus, these DON metabolites contained the intact carbon skeleton of DON as part of their molecular structure. No DON degradation products with less than 15 carbon atoms were found. Moreover, all determined masses were different and clearly higher than the molecular weight of DON, indicating the formation of several structurally different DON conjugates by wheat. Figure 1 depicts the extracted ion currents of the non-labelled and ^13^C-labelled metabolite ions representing all 57 features of the detected compounds.

Among the detected substances, the identity of DON (peak #6) and D3G (*m*/*z* 459.1848, [M+H]^+^, peak #7) was confirmed by standard measurements. This result shows that also the susceptible cultivar Remus has the capacity to form D3G [15].

A closer manual inspection of all isotopic patterns of both the ^12^C monoisotopic as well as the fully ^13^C-labelled ions revealed the presence of sulphur in three of the detected DON conjugates. The isotope ^34^S was confirmed by the presence of a mass peak with a relative intensity of about 4 % and a mass difference of +1.9956 Da from the principal ions in the respective spectra. Most importantly, our study identified DON-GSH (*m*/*z* 604.2158, [M+H]^+^, peak #4) to be formed by wheat. [M+Na]^+^ ions for DON-GSH were observed; the molecular mass of the intact metabolite was determined with a mass error of −2.4 ppm. Additionally, two processing products of the glutathione conjugate of DON were found, peak #2 is putatively identified as DON-*S*-cysteine (−3.2 ppm) and peak #3 was annotated as DON-*S*-cysteinyl-glycine (−2.2 ppm).

In addition to D3G and the glutathione-related conjugates, five currently unknown DON-biotransformation products (peaks #1, #5, #8, #9 and #10) were found. For a more detailed structural characterisation of the detected DON-GSH, MS/MS measurements using collision-induced dissociation were carried out.

### Characterisation of DON-GSH, DON-*S*-cysteinyl-glycine and DON-*S*-cysteine by LC-MS/MS measurements

**Fig. 2:**
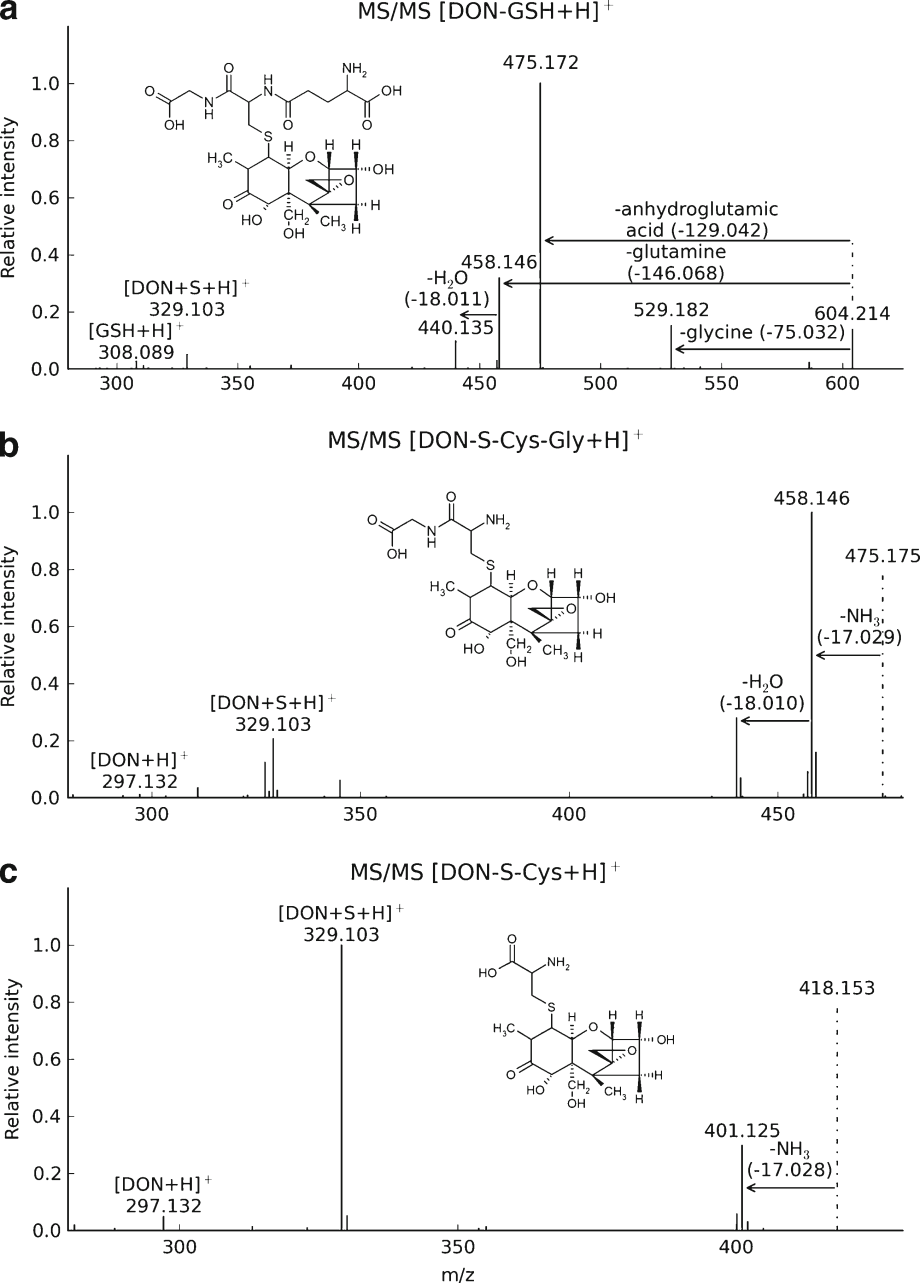
CID MS/MS spectra of DON-GSH (precursor ion, *m/z* 604.21), DON-S-Cys-Gly (precursor ion, *m/z* 475.18) and DON-S-Cys (precursor ion, *m/z* 418.15) with suggested structure formula

Further, MS/MS measurements of DON-treated wheat using the precursor mass *m*/*z* 604.21 (corresponding to [DON-GSH+H]^+^) at 18 eV revealed the characteristic fragmentation behaviour of GSH adducts described by Levsen et al. [20]. Losses of glycine (75 Da), anhydroglutamic acid (129 Da), glutamine (146 Da), as well as the cleavage of the S-CH_2_ bond (275 Da) and DON (296 Da) for DON-GSH were observed (Fig. 2a). For structure confirmation of DON-*S*-Cys-Gly and DON-*S*-Cys, further MS/MS measurements of the precursor ions *m*/*z* 475.18 (at 25 eV) and *m*/*z* 418.15 (at 22 eV) have been carried out respectively (Fig. 2b, c). For both DON conjugates, the MS/MS spectra contained an abundant loss of NH_3_ (17 Da), the cleavage of the S-CH_2_ bond and [DON+H]^+^ which is in good agreement with the characteristic MS/MS fragments of GSH conjugates as described earlier [20].

Previously, only D3G has been reported in the literature as detoxification product of DON *in planta* [21]. Based on the transcriptome response of barley after DON treatment, the formation of DON-GSH conjugates has been postulated, and the non-enzymatic formation of a complex mixture of glutathione conjugates has been demonstrated by NMR [22]. To our best knowledge, our work reports the formation of DON-GSH *in planta* for the first time. The addition of GSH to the double bond of DON, for which NMR evidence was provided [22], seems most likely and therefore this structure (without consideration of its stereochemistry) is shown in Fig. 2. The formation of the glutathione adducts with α,β-unsaturated ketones is a reversible reaction [23], effectively preventing their isolation. Moreover, the occurrence of the glutathione conjugate processing products DON-*S*-Cys-Gly and the DON-*S*-Cys conjugate in wheat are described here for the first time. Our data are in good agreement with well-known detoxification reactions of xenobiotics such as herbicides *in planta* via GSH conjugation and subsequent hydrolysis of the formed conjugates in the vacuole of plant cells [24]. Furthermore, our results do also fit to the observation in barley, where DON treatment triggered a strong upregulation of cysteine biosynthesis suggesting that cysteine is used for glutathione formation and conjugation to DON [22]. The relevance of DON-GSH and its GSH-related biotransformation products regarding food safety is currently unknown and has to be investigated in future studies. Furthermore, additional studies will be needed to elucidate the structure of the remaining, currently unknown DON conjugates. These preliminary results show the high potential of the presented approach, which can be used to study the metabolisation of all types of xenobiotics independent of the nature of the biological system.

## Conclusion

The presented SIL-assisted metabolomics approach in combination with LC-HRMS is a powerful tool for the untargeted screening of biotransformation products of xenobiotics in virtually all types of biological systems. Labelling-specific isotopic patterns can be reliably and automatically detected after measurement of biological samples treated with a 1 + 1 mixture of labelled and non-labelled precursors. Moreover, the data patterns directly reveal the number of carbon atoms in the detected metabolite ions. In this preliminary study, the great potential of the presented approach is further underlined by the successful and automated detection of eight novel plant-derived biotransformation products of the mycotoxin DON. The detection of the DON-GSH conjugate and derived processing products in wheat is reported for the first time, providing evidence for glutathione-mediated metabolism and presumably detoxification of the *Fusarium* virulence factor DON *in planta*.
